# Comparative Evaluation of Consumer Wearable Devices for Atrial Fibrillation Detection: Validation Study

**DOI:** 10.2196/65139

**Published:** 2025-01-09

**Authors:** Femke Wouters, Henri Gruwez, Christophe Smeets, Anessa Pijalovic, Wouter Wilms, Julie Vranken, Zoë Pieters, Hugo Van Herendael, Dieter Nuyens, Maximo Rivero-Ayerza, Pieter Vandervoort, Peter Haemers, Laurent Pison

**Affiliations:** 1 Limburg Clinical Research Center/Mobile Health Unit, Faculty of Medicine and Life Sciences, Hasselt University, Hasselt, Belgium; 2 Department Future Health, Ziekenhuis Oost-Limburg, Genk, Belgium; 3 Department of Cardiology, Ziekenhuis Oost-Limburg, Genk, Belgium; 4 Department of Cardiovascular Sciences, KU Leuven, Leuven, Belgium; 5 Data Science Institute, Hasselt University, Hasselt, Belgium

**Keywords:** atrial fibrillation, AF, mobile health, photoplethysmography, electrocardiography, smartphone, consumer wearable device, wearable devices, detection, electrocardiogram, ECG, mHealth

## Abstract

**Background:**

Consumer-oriented wearable devices (CWDs) such as smartphones and smartwatches have gained prominence for their ability to detect atrial fibrillation (AF) through proprietary algorithms using electrocardiography or photoplethysmography (PPG)–based digital recordings. Despite numerous individual validation studies, a direct comparison of interdevice performance is lacking.

**Objective:**

This study aimed to evaluate and compare the ability of CWDs to distinguish between sinus rhythm and AF.

**Methods:**

Patients exhibiting sinus rhythm or AF were enrolled through a cardiology outpatient clinic. The participants were instructed to perform heart rhythm measurements using a handheld 6-lead electrocardiogram (ECG) device (KardiaMobile 6L), a smartwatch-derived single-lead ECG (Apple Watch), and two PPG-based smartphone apps (FibriCheck and Preventicus) in a random sequence, with simultaneous 12-lead reference ECG as the gold standard.

**Results:**

A total of 122 participants were included in the study: median age 69 (IQR 61-77) years, 63.9% (n=78) men, 25% (n=30) with AF, 9.8% (n=12) without prior smartphone experience, and 73% (n=89) without experience in using a smartwatch. The sensitivity to detect AF was 100% for all devices. The specificity to detect sinus rhythm was 96.4% (95% CI 89.5%-98.8%) for KardiaMobile 6L, 97.8% (95% CI 91.6%‐99.5%) for Apple Watch, 98.9% (95% CI 92.5%‐99.8%) for FibriCheck, and 97.8% (95% CI 91.5%‐99.4%) for Preventicus (*P*=.50). Insufficient quality measurements were observed in 10.7% (95% CI 6.3%-17.5%) of cases for both KardiaMobile 6L and Apple Watch, 7.4% (95% CI 3.9%‐13.6%) for FibriCheck, and 14.8% (95% CI 9.5%‐22.2%) for Preventicus (*P*=.21). Participants preferred Apple Watch over the other devices to monitor their heart rhythm.

**Conclusions:**

In this study population, the discrimination between sinus rhythm and AF using CWDs based on ECG or PPG was highly accurate, with no significant variations in performance across the examined devices.

## Introduction

Atrial fibrillation (AF) is the most prevalent cardiac arrhythmia among adults and is associated with an increased risk of stroke and mortality [1-4]. Since AF is often asymptomatic and paroxysmal, its diagnosis can be challenging. Asymptomatic AF frequently remains undetected until a thromboembolic event occurs. The diagnosis of AF requires an electrocardiogram (ECG) showing irregular R-R intervals (when atrioventricular conduction is not impaired) and the absence of distinct repeating P waves [4].

Historically, ECG diagnostics were confined to medical settings, using devices administered or prescribed by health care professionals. However, the 21st century has witnessed a rapid surge in the availability and use of consumer-oriented wearable devices (CWDs) capable of monitoring heart rhythm [5-8]. Although this trend represents a significant democratization of access to heart rhythm monitoring, it also presents unique challenges for practicing clinicians to diagnose AF based on CWD data, due to the heterogeneous technologies and evolving algorithms used by these devices [9].

The underlying CWD technology is mainly based on electrocardiography or photoplethysmography (PPG). While an ECG measures electrical signals from the heart using surface electrodes on the skin, PPG analyzes the heart rhythm through an optical technique that measures the peripheral pulse [10]. This PPG technology has been adopted by smartwatches and smartphones, using light-emitting diodes and sensors or smartphone flashlights and cameras to generate PPG waveforms. The CWDs analyze ECG or PPG measurements using proprietary algorithms to notify the users of potential AF episodes [9,11,12]. However, only a few CWDs have received medical certification from the US Food and Drug Administration or the Conformité Européenne mark [13].

The emergence of CWDs equipped with automatic heart rate analysis presents a significant opportunity for the early detection of AF. These devices empower patients to monitor their heart rhythms independently, beyond traditional health care settings or without the need for physicians’ prescriptions [9,14-16]. This shift enables more frequent and timely heart rhythm assessments, potentially leading to the earlier identification of AF episodes. However, this democratization of health monitoring also implies that physicians have limited involvement in selecting devices used by patients for heart rhythm analysis. Consequently, it becomes imperative for health care providers to adeptly interpret data from CWDs and assess the reliability of automated rhythm classifications of these devices. These skills are crucial to counsel patients who receive AF detections from CWDs effectively, especially as patients increasingly seek guidance on the accuracy of CWD measurements and on selecting the most suitable device.

Despite numerous validation studies of individual CWDs, a comprehensive comparison of their methodologies and outcomes is challenging, which leaves a gap in our understanding of their comparative effectiveness. This study aims to bridge this knowledge gap by evaluating and comparing the ability of digital CWDs to classify heart rhythms, specifically focusing on their accuracy in distinguishing between sinus rhythm and AF compared to the gold standard 12-lead ECG.

## Methods

### Study Design

Patients were recruited from July to November 2023. The VALIDATION (Validation of Digital Heart Rhythm Devices in the Detection of Atrial Fibrillation) study was a monocentric, prospective, randomized, interventional study that validated the use of PPG- and ECG-based CWDs to detect AF compared to a simultaneous 12-lead ECG in ambulatory cardiology patients in a supervised hospital environment.

### Ethical Considerations

The study protocol is in accordance with the Declaration of Helsinki and the International Council for Harmonisation of Technical Requirements for Pharmaceuticals for Human Use – Good Clinical Practice guidelines and was approved by 2 medical ethics committees (ie, Ziekenhuis Oost-Limburg, Genk, Belgium, Z-2023023; and Hasselt University, Hasselt, Belgium, B3712023000006). The study was registered at ClinicalTrials.gov (NCT06023290) and meets all STARD 2015 criteria. Informed consent was obtained in writing from all participants. Moreover, they were informed about their right to opt out of the study at any time without consequences. Data collected during the study was pseudonymized to protect participant confidentiality. Unique identifiers were used, and direct personal information was stored separately, accessible only to authorized personnel. Participants were not compensated for their involvement in the study as participation did not require an additional hospital visit and involved minimal additional time or effort.

### Study Population

The study employed two distinct methods for participant inclusion, targeting adult patients from the cardiology outpatient clinic (Multimedia Appendix 1). First, all patients visiting the outpatient cardiology clinic were screened by chart review for eligibility on selected days when study personnel were available. Second, to enrich the study population with a higher prevalence of AF, all patients scheduled for electrical cardioversion at the cardiology outpatient clinic were also identified for screening by chart review.

Patients who met the following inclusion and exclusion criteria were invited to participate in the study: (1) age 18 years or older and (2) exhibit sinus rhythm or AF. Exclusion criteria were (1) presence of a pacemaker, (2) participation in another clinical trial that might interfere with the study protocol, and (3) physical or cognitive restrictions, including language barriers that hindered study measurements.

### Study Measurements

After the participants provided informed consent, they were instructed by study personnel to perform heart rhythm measurements using 4 CWDs in a random sequence consisting of 2 ECG- and 2 PPG-based devices, according to the manufacturer’s instructions. During all measurements, the patients were also instructed to stay still to avoid motion artifacts. All CWDs were provided by the study team to prevent device-induced bias.

KardiaMobile 6L (AliveCor Inc, Mountain View, CA) and a smartwatch, Apple Watch (Apple Inc, Cupertino, CA), were used to derive a single-lead ECG of 30 seconds. A lead-1 ECG was recorded using KardiaMobile 6L by positioning the device on the left knee with both thumbs on the device’s front electrodes [17]. To capture a lead-1 ECG using the Apple Watch, the participant wore the watch on the left wrist, ensuring skin contact with the electrode on the watch’s underside. Measurement was initiated by the participant by touching a second electrode on the watch’s crown with the right hand’s index finger.

The PPG measurements were performed with two smartphone apps: (1) FibriCheck (Qompium NV, Hasselt, Belgium) and (2) Preventicus Heartbeats App (Preventicus GmbH, Jena, Germany). While initiating a PPG measurement in either smartphone app, the camera’s flashlight was activated. Participants were instructed by study personnel to place an index finger over the smartphone camera. Using the camera, the app then captured skin color variations caused by heartbeat-induced blood flow changes. These variations were analyzed by the app to generate the PPG waveform.

The final rhythm diagnosis was made by the device’s proprietary algorithm for both ECG and PPG measurements. Although 6 leads were measured using the KardiaMobile 6L device, its algorithm only took lead 1 into account. To minimize the occurrence of missing data, measurements were repeated up to 3 attempts until a good-quality measurement was obtained with each device. If good-quality measurements could not be obtained with a specific device, the patient’s results were not excluded from the analyses. Good quality was defined as sufficient quality for analysis by the device algorithm. Rhythm diagnosis from CWDs were provided by the proprietary device algorithms and categorized as sinus rhythm or AF (Textbox 1). Concurrently with the CWD measurements, a continuous 12-lead ECG was performed for reference monitoring (MAC5000, GE Healthcare, Chicago, IL). Participants were excluded from the analyses if the heart rhythm changed on the reference ECG during the study conduct. The reference diagnosis on the 12-lead ECG was provided by two cardiologists blinded to the CWD classifications and the participant’s clinical information.

Textbox 1. **Categorization of consumer-oriented wearable device outcome labels.**

**Categorized as sinus rhythm**

-  KardiaMobile 6L

-   Normal
-   Bradycardia
-   Tachycardia

-  Apple Watch

-   Sinus rhythm
-   High heart rate - no atrial fibrillation
-   Low or high heart rate

-  FibriCheck

-   Normal
-   Mild irregularities

-  Preventicus

-   Normal
-   Mild irregularities

**Categorized as atrial fibrillation**

-  KardiaMobile 6L

-   Possible atrial fibrillation

-  Apple Watch

-   Atrial fibrillation
-   Atrial fibrillation - high heart rate

-  FibriCheck

-   Possible atrial fibrillation

-  Preventicus

-   Possible atrial fibrillation

**Categorized as insufficient quality**

-  KardiaMobile 6L

-   No analysis
-   Unclassified
-   Unreadable

-  Apple Watch

-   Inconclusive
-   Poor reading

-  FibriCheck

-   Insufficient quality

-  Preventicus

-   Insufficient quality

### Study Questionnaire

Following the completion of study measurements, participants were provided a 5-point Likert scale questionnaire designed to assess their experiences with and preferences regarding the devices used in the study. Additionally, the questionnaire aimed to evaluate the participants’ digital literacy levels prior to their involvement in the study.

### Data Analysis

Data analysis was performed using SPSS software (version 29.0; IBM Corp) and SAS (version 9.4; SAS Institute). *P*<.05 was considered statistically significant. The Shapiro-Wilk statistic was used to assess the normality of continuous data. Continuous variables are presented as median and IQR as appropriate and were compared using the Mann-Whitney *U* test. Discrete variables are presented as absolute numbers and percentages (%) and compared using Pearson *χ*^*2*^ test. The performance of the CWDs were evaluated by calculating their sensitivity, specificity, and accuracy compared to the 12-lead reference ECG. These metrics were estimated and compared between CWDs using a generalized estimation equation model, accounting for the correlated nature of the measurements of the same patient. Measurements of insufficient quality were not included in these analyses.

## Results

### Overview

On designated study days, 200 patients (145 attending outpatient consultations and 55 undergoing elective cardioversions) were invited to participate in the study. Among these, 123 subjects were included, of which 122 completed the study protocol (see Multimedia Appendix 1). The remaining 77 patients either declined participation due to a lack of interest or were unwilling to commit the additional time required for study procedures. One participant with paroxysmal AF, who exhibited both sinus rhythm and AF on the reference ECG during the study, was excluded from the analysis to avoid confounding results. This decision was made because the specific rhythm present during the CWD recordings could not be reliably determined.

### Study Population Characteristics

**Figure 1.**
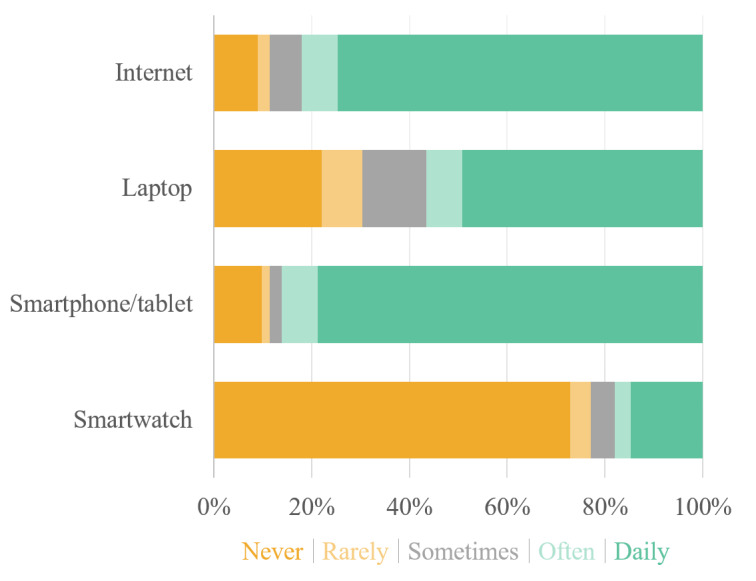
Experience with digital technology. Questions: I use the internet; I use a computer and/or laptop; I use a smartphone and/or tablet; I use a wearable device (eg, fitness tracker, smartwatch).

The median age of the study population was 69 (IQR 61-77) years, with 44 of the 122 (36.1%) participants being women. All participants were White. Detailed demographic and clinical characteristics are given in Table 1, and information on drug therapy is provided in Multimedia Appendix 2. There were no adverse events related to the use of the CWDs. Based on the 12-lead reference ECG, AF was present in 30 (24.6%) patients. The majority of the study population demonstrated a fair level of digital literacy, with 91 (74.6%) participants reporting daily internet usage and 96 (78.7%) using smartphones daily, respectively, as illustrated in Figure 1. However, 16 (13.1%) participants were identified as potentially digitally illiterate, having no prior experience with the internet or a smartphone. Furthermore, 89 (73%) participants reported no previous experience with CWDs, except smartphones.

**Table 1.** Demographic and clinical characteristics of the study population.

Participant characteristics	Participants			*P* value^a^
	Overall (n=122)	SR^b^ (n=92)	AF^c^ (n=30)	
Age (years), median (IQR)	69 (61-77)	68 (60.25‐77.0)	72.5 (63.5‐78.5)	.17
Gender (women), n (%)	44 (36.1)	34 (37)	10 (33.3)	.72
BMI (kg/m^2^), median (IQR)	26.9 (24.1‐30.8)	26.9 (23.5‐30.3)	28.2 (25.7‐33.6)	.02
Hypertension, n (%)	69 (56.6)	51 (55.4)	18 (60)	.66
Diabetes, n (%)	21 (17.2)	17 (18.5)	4 (13.3)	.52
Hypercholesterolemia, n (%)	83 (68)	65 (70.7)	18 (60)	.28
Heart failure, n (%)	21 (17.2)	12 (13)	9 (30)	.03
Vascular disease history, n (%)	26 (21.3)	22 (23.9)	4 (13.3)	.22
Stroke, n (%)	19 (15.6)	15 (16.3)	4 (13.3)	≥.99
CHA_2_DS_2_-VASc scores^d^, n (%)				.50
0	12 (9.8)	9 (9.8)	3 (10)	
1	22 (18)	16 (17.4)	6 (20)	
2	21 (17.2)	18 (19.6)	3 (10)	
3	33 (27)	23 (25)	10 (33.3)	
4	21 (17.2)	18 (19.6)	3 (10)	
5	6 (4.9)	3 (3.3)	3 (10)	
6	7 (5.7)	5 (5.4)	2 (6.7)	

a Chi-square test, Fisher exact test, and Mann-Whitney *U* test were used in the analyses.

b SR: sinus rhythm.

c AF: atrial fibrillation.

d CHA_2_DS_2_-VASc is a points-based system used to stratify the risk of stroke in patients with AF. CHA_2_DS_2_-VASc is calculated as congestive heart failure, hypertension, age ≥75 years (doubled), diabetes, stroke (doubled), vascular disease, aged 65-74 years, and sex category (female) [18].

### Signal Quality

**Figure 2.**
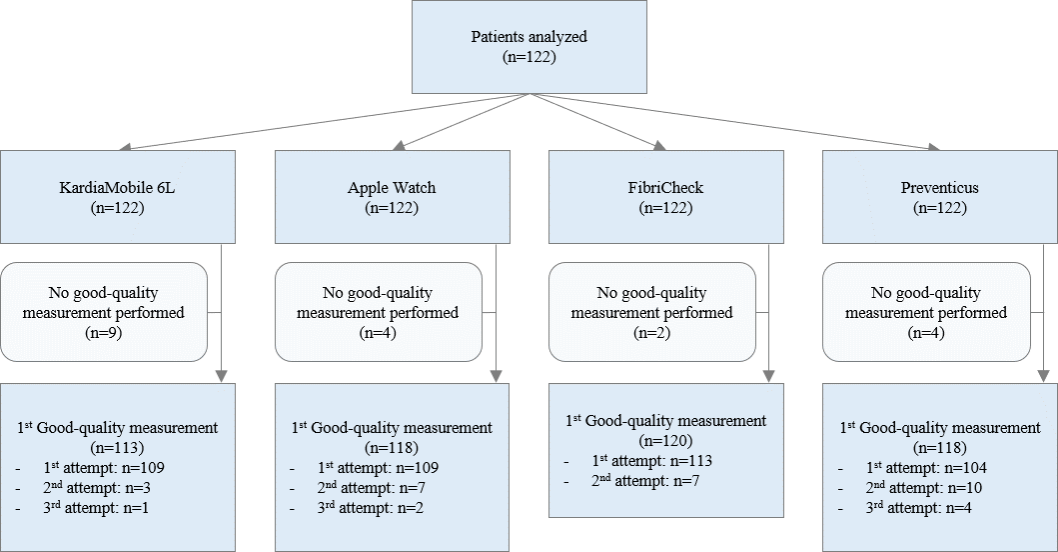
Signal quality for each digital device. The flowchart describes the number of patients in which no good-quality measurement could be obtained after three attempts and the number of attempts necessary to obtain a good-quality measurement for each device.

Measurements of insufficient quality were repeated until the CWD algorithm provided a rhythm diagnosis. The number of additional measurements required for diagnosis is illustrated in Figure 2. Good-quality measurements could not be obtained after three attempts in 9 (7.4%) patients with KardiaMobile 6L, 4 (3.3%) with Apple Watch, 2 (1.6%) with FibriCheck, and 4 (3.3%) with Preventicus. Achieving good-quality measurements required 5 additional attempts for KardiaMobile 6L, 11 for Apple Watch, 7 for FibriCheck, and 18 for Preventicus. Consequently, the prevalence of insufficient quality measurements was 10.7% (95% CI 6.3%-17.5%) for both KardiaMobile 6L and Apple Watch, 7.4% (95% CI 3.9%-13.6%) for FibriCheck, and 14.8% (95% CI 9.5%-22.2%) for Preventicus, with no significant differences between the devices (*P*=.21). Furthermore, the distribution of insufficient quality measurements was comparable between patients in sinus rhythm and AF (11.7% vs 8.3%, *P*=.31).

Patients with insufficient quality recordings from both ECG and PPG devices were significantly older, with a median age of 83 (IQR 77-86) years compared to 68 (IQR 60-76) years (*P*<.001). However, there was no significant age difference between patients with insufficient quality recordings from either ECG or PPG devices and those with only good-quality recordings with a median age of 71 (IQR 62-80) years and 68 (IQR 60-75) years, respectively (*P*=.14). Similarly, no significant age difference was observed among patients with insufficient quality recordings (median age 74, IQR 62-80 years) versus those with only good-quality recordings (median age 68, IQR 60-75 years) from PPG devices specifically (*P*=.14). For ECG devices, measurements of insufficient quality were more often performed by older patients with a median age of 74 (IQR 64-82) years versus 68 (IQR 60-75) years (*P*=.02). Despite reduced digital literacy in older patients (*P*<.001), there was no significant association between digital literacy and the occurrence of insufficient data quality from either ECG- or PPG-based CWDs (*P*=.14).

### Heart Rhythm Classification

Based on the first good-quality measurement, all patients with AF were correctly identified by all CWDs (Table 2). Therefore, the sensitivity for all CWDs to detect AF was 100%. The specificity rates for identifying sinus rhythm were 96.4% for KardiaMobile 6L, 97.8% for Apple Watch, 98.9% for FibriCheck, and 97.8% for Preventicus. The differences in specificity between devices were not statistically significant (*P*=.50). The diagnostic performance of all study devices is detailed in Table 3.

**Table 2.** Cross-tabulation of the consumer-oriented wearable device results compared to the 12-lead reference electrocardiogram (ECG).

	12-lead ECG rhythm (n=113)	
	SR^a^	AF^b^
KardiaMobile 6L, n/N^c^ (%)		
SR	81/84 (96.4)	0/29 (0)
AF	3/84 (3.6)	29/29 (100)
Apple Watch, n/N (%)		
SR	89/91 (97.8)	0/27 (0)
AF	2/91 (2.2)	27/27 (100)
FibriCheck, n/N (%)		
SR	89/90 (98.9)	0/30 (0)
AF	1/90 (1.1)	30/30 (100)
Preventicus, n/N (%)		
SR	87/89 (97.8)	0/29 (0)
AF	2/89 (2.2)	29/29 (100)

a SR: sinus rhythm.

b AF: atrial fibrillation.

c N represents the number of detections by the 12-lead reference ECG for the SR and AF columns.

**Table 3.** Comparison of performance of the consumer-oriented wearable devices compared to the 12–lead reference electrocardiogram.^a^

Performance metrics	KardiaMobile 6L^b^	Apple Watch^b^	FibriCheck^c^	Preventicus^c^	*P* value
Good-quality tracing, % (95% CI)	89.3 (82.5-93.7)	89.3 (82.5-93.7)	92.6 (86.4-96.1)	85.3 (77.8-90.5)	.21
Sensitivity, % (95% CI)	100	100	100	100	—^d^
Specificity, % (95% CI)	96.4 (89.5-98.8)	97.8 (91.6-99.5)	98.9 (92.5-99.8)	97.8 (91.5-99.4)	.50
Accuracy, % (95% CI)	97.4 (92.1-99.1)	98.3 (93.5-99.6)	99.2 (94.3-99.9)	98.3 (93.5-99.6)	.48

a Generalized estimation equation model was used for the analysis.

b Single-lead electrocardiogram (lead 1).

c Smartphone photoplethysmography (finger over the camera).

d Not applicable.

### Study Questionnaire

**Figure 3.**
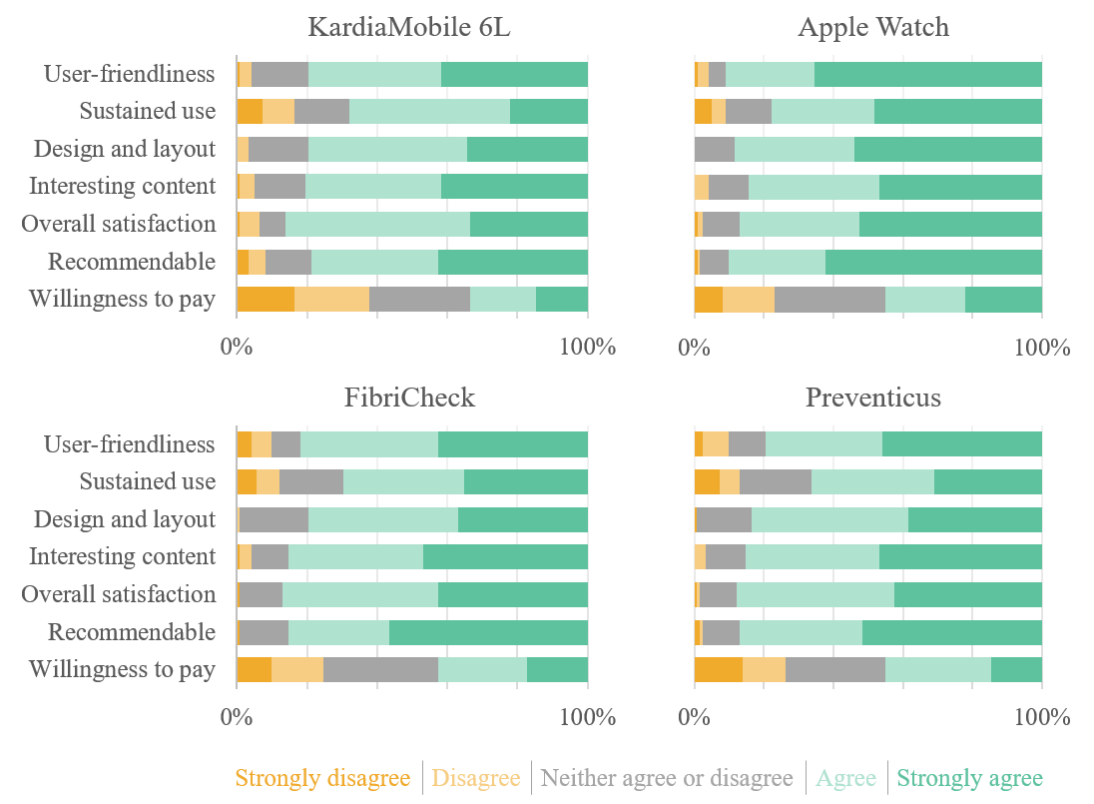
Study questionnaire responses. Questions: This device / app was easy to use; I would use this device / app again; I like the design and layout of this device / app; the content of this device / app is interesting to me; in general, I am satisfied with this device / app; I would recommend this device / app to people who would benefit from it; I would pay for this device / app (no price disclosed).

After performing at least two measurements with each CWD, a significant majority of participants (over 80%) rated the study devices favorably across various criteria, including user-friendliness, likelihood of sustained use, design and layout, interesting content, overall satisfaction, and likelihood of recommending the devices to others (Figure 3). In these evaluations, the Apple Watch scored higher on the questionnaire, followed by FibriCheck, Preventicus, and KardiaMobile 6L. Despite these positive assessments, cost emerged as the principal barrier to broader adoption and implementation of these devices.

## Discussion

### Principal Findings

This study validated the ability of four CWDs to differentiate between sinus rhythm and AF using ECG or PPG technology and their proprietary algorithms. The devices were compared against a concurrently obtained 12-lead reference ECG, demonstrating very high sensitivity and specificity, thus confirming a near-perfect association with the diagnosis made using the 12-lead reference ECG.

To our knowledge, this is the first study to directly compare the accuracy of automated AF detection by ECG- and PPG-based CWDs against simultaneous 12-lead reference ECG monitoring. These findings align with prior studies that separately validated PPG and single-lead ECG CWDs [17,19-21]. However, the exclusion of patients and handling of uninterpretable results can significantly impact outcomes and hamper comparisons between studies [17]. A meta-analysis of 28 validation studies of PPG-based CWDs revealed significant biases, mainly concerning participant selection and insufficient information on study flow, design, and timing [19].

Addressing the limitations of previous studies, this trial adopted an inclusive approach by including all patients with a scheduled visit on designated study days, conducting simultaneous reference and index tests, and randomizing the sequence of index tests to minimize biases. However, a large number of the patients could not be screened or did not meet inclusion criteria, limiting findings to a specific demographic of adult White patients without a pacemaker in AF or sinus rhythm without frequent extrasystoles, who were capable of performing CWD measurements. Unlike previous studies, which have shown a greater incidence of uninterpretable measurements in up to one-third of cases, this study limited exclusions to 7.4%-14.8% by iterating measurements until a diagnosis was obtained. These measurements were excluded from this validation study, leading to an overestimation of the algorithm’s accuracy [17,21,22]. This approach reflects real-world use, where users typically repeat measurements until a diagnosis is obtained from the device. Older patients, expectedly, were more likely to produce recordings of insufficient quality, frequently due to tremors.

Future validation studies are needed to validate these results in non-White populations and in individuals with rhythms other than sinus rhythm or AF, or with frequent extrasystoles that may challenge the algorithm’s specificity. In our study, false-positive results from both ECG- and PPG-based CWDs were more likely when the 12-lead reference ECG showed premature atrial or ventricular contractions. These findings suggest that frequent extrasystoles can lead to rhythm misclassifications, highlighting the need for further research into how these irregularities affect device performance, particularly in populations with diverse arrhythmias or more frequent extrasystoles. The study population included patients with both high and low digital literacy, which did not affect their performance in a supervised hospital environment. However, CWDs are mostly used in unsupervised settings. Recent real-world validation studies in unsupervised environments have exhibited similar results [23,24].

This study has implications for clinical practice as it provides physicians with information about the accuracy of CWDs. Physicians are likely to encounter CWDs, either by the patient’s choice or owing to these devices being marketed and sold directly to the consumer. This comparative validation study of several devices with heterogeneous technologies and algorithms enables physicians to counsel patients on measurement results or the selection of medically certified devices. Similar studies should be repeated over time as algorithms are frequently updated, potentially improving diagnostic accuracy.

Given the high prevalence of uninterpretable device ECGs, mass utilization by the general population may lead to an increased and unnecessary health care burden [25]. To support clinical decisions based on CWD heart rhythm measurements, physicians should not rely solely on algorithm results, instead they should interpret traces manually when required. Although manual interpretation of CWDs was not within the scope of this study, it was investigated in the INTERPRET-AF study which reported similar accuracy for PPG- and ECG-based CWDs upon interpretation by physicians, with only modest reductions compared to the 12-lead ECG, even without prior PPG training [26].

The potential of CWDs in detecting new AF cases in high-risk populations (eg, secondary prevention in cryptogenic stroke patients) or monitoring AF recurrence in patients with an established diagnosis highlights the evolving landscape of cardiac care [23,27]. Despite increased AF detection and cost-effectiveness of these implementations, the stroke risk and therapeutic implications for AF detected by screening remain debatable [28]. Patients’ willingness to adopt these CWDs was reported in over 80% of the questionnaire responses, signaling an increasing likelihood of implementation in the future.

### Limitations

This study has several limitations. First, tracings deemed to be of insufficient quality were excluded from the analyses, potentially overestimating the performance and influencing the generalizability of the results. Second, patients with frequent extrasystoles or rhythms other than sinus rhythm or AF, as documented in their medical records, were excluded from this study, which could have reduced the specificity. Nevertheless, extrasystoles that occurred during the study procedures were included in the analyses, leading to false positive findings. The results of the CWD’s algorithms were categorized into only two categories (ie, sinus rhythm and AF), without accounting for a broader range of differential diagnoses. Third, participants received instructions on device usage and were corrected when necessary to optimize signal quality. Fourth, this study could not evaluate the effect of skin color on the diagnostic accuracy of these devices. Fifth, the algorithm of the CWD with multiple ECG leads (ie, KardiaMobile 6L), which may have increased the specificity, was evaluated using 1 lead [17].

### Conclusions

In this selected population, the distinction between sinus rhythm and AF using CWDs based on ECG or PPG was highly accurate, with no discernible variations observed across the examined devices.

## Supplementary material

10.2196/65139 Multimedia Appendix 1 Flowchart of study screening and inclusion. AF: atrial fibrillation; SR: sinus rhythm; SVES: supraventricular extrasystoles; VES: ventricular extrasystoles.

10.2196/65139 Multimedia Appendix 2 Medication use of the study population. Chi-square test was used in the analyses. ACE: angiotensin-converting enzyme; AF: atrial fibrillation; ARB, angiotensin receptor blockers; CCB: calcium channel blockers; OAC: oral anticoagulation; SR: sinus rhythm.

## Abbreviations

AF: atrial fibrillation
CWD: consumer-oriented wearable device
ECG: electrocardiogram
PPG: photoplethysmography
VALIDATION: Validation of Digital Heart Rhythm Devices in the Detection of Atrial Fibrillation
