# Supplementary figures and images for: Comparative Evaluation of Consumer Wearable Devices for Atrial Fibrillation Detection: Validation Study
Source: JMIR Form Res. 2025 Jan 9;9:e65139. doi: 10.2196/65139 (PMC11737281; doi:10.2196/65139)

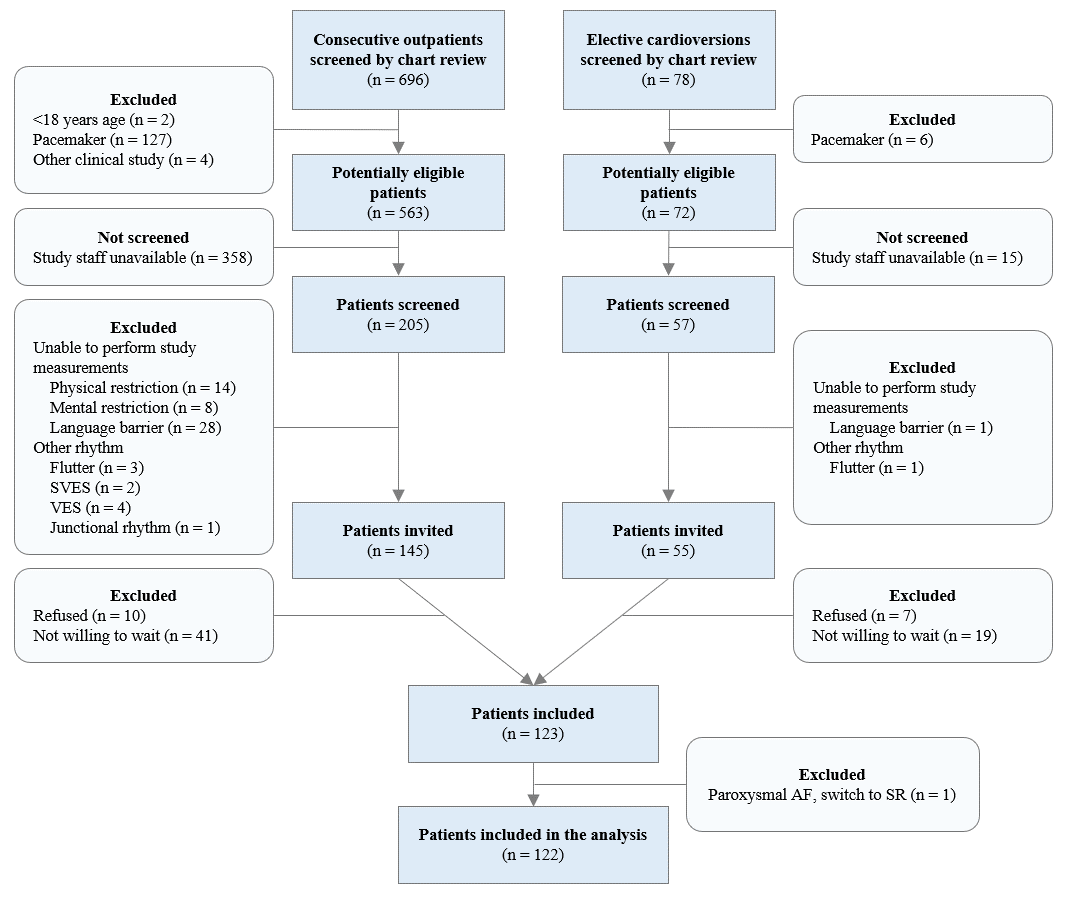

Supplement: Multimedia Appendix 1 [file formative-v9-e65139-s001.png]

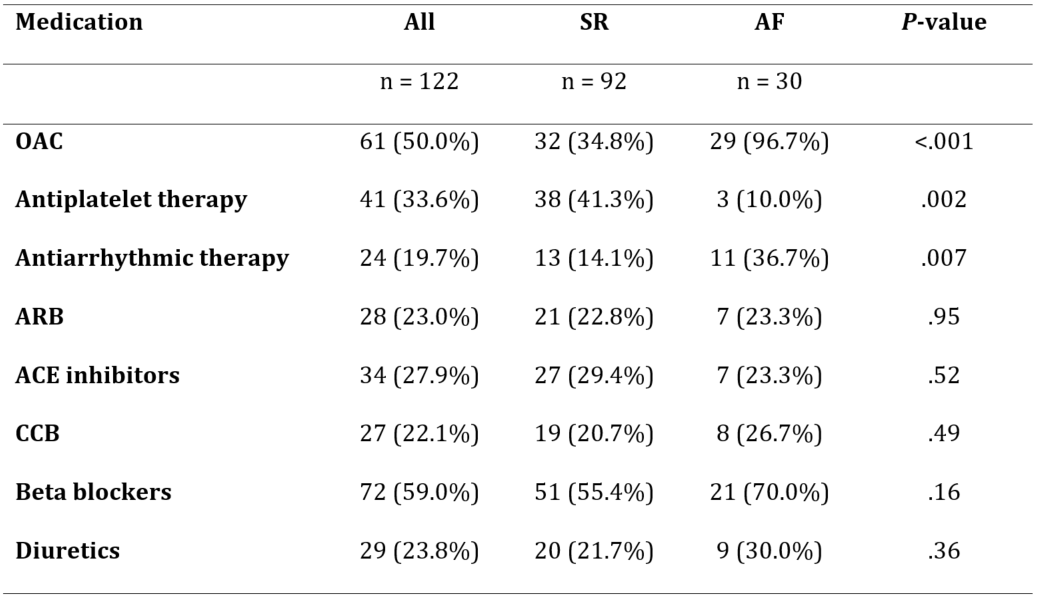

Supplement: Multimedia Appendix 2 [file formative-v9-e65139-s002.png]
